# The suppression of spontaneous face touch and resulting consequences on memory performance of high and low self-touching individuals

**DOI:** 10.1038/s41598-022-12044-4

**Published:** 2022-05-23

**Authors:** Jente L. Spille, Martin Grunwald, Sven Martin, Stephanie M. Mueller

**Affiliations:** Haptic Research Laboratory, Paul Flechsig Institute – Centre of Neuropathology and Brain Research, University of Leipzig, 04103 Leipzig, Germany

**Keywords:** Psychology, Human behaviour

## Abstract

Spontaneous touching of one's own face (sFST) is an everyday behavior that occurs primarily in cognitively and emotionally demanding situations, regardless of a persons’ age or gender. Recently, sFST have sparked scientific interest since they are associated with self-inoculation and transmission of respiratory diseases. Several studies addressed the need to reduce sFST behaviors without discussing the underlying functions of this spontaneous behavior. In addition, the question of why this behavior occurs very frequently in some individuals (high self-touching individuals, HT) but less frequently in others (low self-touching individuals, LT) has not yet been addressed. For the first time, we distinguished between HT and LT and investigated the behavioral consequences of sFST suppression in these two groups. For this purpose, we examined performance outcomes of 49 participants depending on sFST behaviors during a haptic working memory task. In addition, we assessed personality traits of HT and LT using the Freiburg Personality Inventory (FPI-R). The results of our study reveal that suppressing sFST in HT is negatively related to memory performance outcomes. Moreover, HT show tendencies to differ from LT in certain personality traits. Our results highlight the relevance of distinguishing between HT and LT in future studies of sFST.

## Introduction

The high mortality rate of COVID-19 is currently leading to increased research on various aspects of infection prevention, including suppression of spontaneous facial touch. In this context, spontaneous face-touches are of research interest because they are associated with self-inoculation. When hands touch contaminated surfaces or, via handshake, others people’s hands and subsequently touch the own face, pathogens can be transferred to one's own facial mucous membranes^1–3^. Although one study showed that the number of spontaneous touches to the own face (sFST) decreased after handshake^4^, the risk of autoinoculation is ubiquitous because sFST also occur in the absence of others^5–7^ and viruses can survive on both surfaces^8,9^ and fingers^10^ for several hours. Spontaneous facial self-touches especially play a central role because no attention is paid to the initiation and execution of sFST by the person performing it, and recall of the behavior is poor^11,12^. Unlike active facial self-touches, sFST have no apparent motivation (e.g. scratching to relieve an itch) and they are not intended to serve communicative functions such as tipping one’s forehead^13^. While there is increasing research on the interplay of tactile and motor processes as well as neural processing with regard to active self-touches^14–17^, to date there is a lack of research approaches that investigate these processes in sFST.

### Spontaneous facial self-touches

Spontaneous face touching is an everyday behavior that occurs in people of all ages, regardless of sex or ethnicity, up to 800 times during 16 waking hours/day^13^. The behavior involves spontaneously touching one's own face with one or both hands. Studies indicated that sFST occur both during social interaction^18,19^ and in the absence of others^5–7^. The mean duration of sFST has been reported to be less than 3 s in some studies^6,20,21^ and less than 6 s in others^18,22–26^. Most face touches are directed to the midline of the face^13,27^. The sFST behavior occurs more frequently when negative emotions such as anxiety, tension, discomfort, or uncertainty are evoked^28–30^. In this regard, researchers have attributed self-regulatory functions to sFST within emotionally demanding situations^5,6,29,31,32^. Furthermore, researchers have discussed the association between sFST and cognitive load and attentional demands. In line with this, our own research group found an increase in sFST when distracting sounds were presented during a delayed memory task^5,6^. Research findings suggested that distracting sounds divert the focus of attention from the memory that should be maintained in a working memory task^33,34^. According to Grunwald et al., sFST are performed as a consequence of such interference^5^. This assumption is supported by the finding that significantly more sFST occurred during the presentation of distracting sounds than in the silences between sounds^6^. Electroencephalographic study results support the hypothesis that sFST serve the regulation of cognitive processes in demanding situations such as working memory tasks^5,35^.

### Interindividual variability in frequency of sFST

Taking previous studies on sFST into account, the wide range in the frequency of sFST behavior is remarkable. Elder and colleagues observed health care workers while performing their usual duties (e.g. front office, medical examination) and recorded a range of 0–105 sFST across individuals during the two-hour investigation period^36^. In another study, medical students underwent 15 min of observation during the university clinic setting^37^. Among female subjects, the authors observed a range of 0–25 sFST in 15 min, extrapolated to 0–100 sFST/h. According to the authors, the sFST frequency was independent of participants’ gender and of wearing glasses^37^. The highest range of interindividual sFST behavior was reported by Kwok and colleagues, who observed medical students during a two-hour lecture. During an average hour the range of observed sFST was 4–153^25^. Despite the observed ranges in the aforementioned investigations, no study has yet distinguished between individuals who rarely exhibit sFST (low self-touching individuals, LT) and individuals who frequently perform sFST (high self-touching individuals, HT). Ralph and colleagues, who studied sFST behavior whilst driving, have first emphasized the high interindividual variability in sFST that could not be explained by age or gender^26^. They have suggested that personality factors may underlie the individual differences and called for intensive research on possible determinants of sFST behavior.

### Personality and sFST

A previous study examined the relationship between nonverbal communication behaviors and the two personality traits extraversion and neuroticism^38^. The authors found that self-touching was positively associated with a teacher’s rating of another person’s neuroticism, while self-reported neuroticism was not related to the touch behavior. Other research findings that examined the impact of sFST on other people indicate that individuals who performed sFST were perceived as more outgoing, dominant, expressive, and interested than people who did not exhibit sFST^22,23^. It is questionable whether these perceived characteristics are actually reflected in manifest personality traits and whether these are associated with the frequency of sFST behavior.

Because insights into the relationship between personality traits and sFST behavior are limited so far, the present study aims to explore whether there are personality traits that are associated with individual sFST behavior. For this purpose, the Freiburg Personality Inventory (FPI-R)^39^ was chosen, a German multidimensional personality inventory that measures personality traits on 12 different scales: life satisfaction, social orientation, performance orientation, shyness, irritability, aggression, stress, physical complaints, health concerns, openness, extraversion, and emotionality. Following the call for intensive research on possible determinants of sFST behavior^26^, the use of the FPI-R is a suitable approach, insofar as a broad range of personality traits is surveyed.

Previous observational studies have investigated sFST behavior in the context of different situations, without discussing personality traits in relation to sFST. For instance, studies have indicated that the frequency of sFST changed within social interactions. As such, a higher number of sFST was observed when participants unexpectedly had to engage in an informal conversation with an unfamiliar interviewer^18^, or when patients talked about emotionally relevant topics with their physicians^40^. Based on these studies, it cannot be concluded whether the experimental situation mainly influenced the sFST behavior or whether the elevated sFST behavior may be explained by stable personality traits such as shyness or emotionality. A higher sFST frequency was also observed during the accomplishment of cognitive tasks requiring the focusing of attention^5,35^. Barroso and colleagues further found that higher numbers of sFST were associated with better performances in a memory task and an attentional task^41^. Again, it is not possible to clarify whether the increase in sFST was due to the cognitive demands of the specific experimental requirements or an interindividual difference in performance orientation.

Because there are currently few findings on the relationship between personality traits and sFST behavior, and study results on external situational factors do not allow a direct inference on personality traits, no predefined hypotheses are stated with regard to the subscales of the FPI-R. We assume that HT differ from LT in their scores on the scales of the FPI-R (hypothesis 1).

### Suppression of sFST

Several research groups have recently investigated approaches to suppress spontaneous touching of one's own face in order to reduce self-infection and the spread of respiratory diseases. Pathogens can be transferred to mucous membranes—mouth, nose, eyes—by touching one's own face after having contacted contaminated surfaces with the own fingers^42^. Since most sFST are directed to the midline of the face^13^, oral and nasal mucosa are particularly relevant as potential transmission routes. Avoiding sFST should contribute to a reduction of these indirect transmission routes^36^.

A number of researchers have discussed that individuals who rate the severity of disease if infected by pathogens as high exhibit reduced sFST behavior. Johnston and colleagues found that perceived severity of infection predicted lower rates of sFST^43^. Similarly, Carrillo-Diaz and colleagues observed that threat perception of COVID-19 was negatively associated with sFST^44^. In contrast, Kwok and colleagues observed a high number of sFST (M = 23 sFST/h) in medical students who had previously attended an infection control course^25^. Another study investigated the prevalence of adherence to preventive measures (e.g. avoiding touching the eyes and nose) in Chinese students during the H1N1 pandemic. 72.3% of those who completed the survey reported not having reduced the frequency of touching their mouths, noses and eyes as compared with the pre-H1N1 period^45^. Accordingly, it is questionable whether education about the risk of infection associated with sFST has a (long-lasting) effect on sFST behavior. The fact that little or no attention is paid to the initiation and performance of sFST^13^ impedes efforts to voluntarily suppress sFST. Elder and colleagues who had observed health care workers found that medical staff who stated they frequently avoided sFST actually touched their face at the same rate as those who reported to only occasionally or rarely avoid sFST^36^.

Physical barriers that prevent contact between finger and facial skin have recently been explored to reduce the risk of infection during the Covid-19 pandemic. In one study, individuals performed less sFST when wearing latex gloves compared to a control situation without gloves^44^. In another study, tapes were used to prevent the execution of arm flexion that precedes sFST. However, taping the extensor side of the elbow did not result in persistent inhibition of sFST behavior^46^. The influence of protective mouth-nose masks on sFST behavior has currently been the subject of controversy in the research community. Some authors found a negative correlation between wearing a mask and sFST frequency^47,48^. In contrast, some studies reported increased tendencies to touch the face while wearing a face mask^49^ and loose mask slipping off the nose that caused more hand contacts with the face^50^. However, other authors did not observe any differences in sFST frequency depending on the wearing of a mask^51^.

Another attempt to reduce sFST behavior involves different control measures. Carrillo-Diaz and colleagues used signs reminding participants not to touch the face and observed a lower incidence of sFST when reminder signs had been introduced compared to when control measures had been absent^44^. Several research groups developed smart wearable devices that provide, for example, vibrotactile or auditory feedback that send a warning signal when the hand moves closer to the face, thus preventing sFST^52–55^. D'Aurizio and colleagues found a reduction in sFST behavior when wearing a wearable device, but data on long-term effectiveness are lacking^53^. Furthermore, when wearing a smartwatch, for example, only those sFST executed with the arm wearing the watch are detected. As there seems to be no difference in the frequency of left-handed and right-handed sFST^13^, wearing a smartwatch would probably fail to capture a large number of sFST.

### Possible consequences of sFST suppression

Although some approaches to suppress or reduce sFST have been shown to be effective in the aforementioned studies, the question of behavioral consequences of suppressing this spontaneous behavior has been neglected until now. It is remarkable how many research groups are currently investigating possibilities to suppress sFST without addressing possible consequences of this suppression, although the underlying psychological mechanisms of sFST are still poorly understood^13^. Following the assumption that sFST serve the regulation of attentional and memory processes^5,6,35,41^, it is important to consider whether suppressing this inherent regulatory mechanism also affects behavioral outcomes. Suppressing this spontaneous behavior may have negative consequences on attention focus or memory performance. In particular, the performance of HT may be negatively affected by suppression of sFST, because HT are restrained in their natural frequent sFST behavior and thus are limited in their regulatory repertoire. With respect to the performance of LT, it may not make any difference whether sFST can be executed or not, since LT rarely perform sFST anyway. Based on these considerations, we want to investigate the behavioral consequences of mechanically suppressed sFST during the retention interval of a haptic working memory task in HT and LT.

We hypothesize that in HT, suppression of sFST during a working memory task (immobilized hands) will cause a decrease in memory performance and therefore be associated with poorer performance outcomes than when sFST can be performed freely (free hands) (Hypothesis 2). For LT, we assume that performance outcomes do not differ between the “immobilized hands” and “free hands” conditions (Hypothesis 3).

## Materials and methods

### Participants

Forty-nine healthy volunteers took part in the experiment (23 female; age: *M* = 25.39 years, *SD* = 3.21; range 20–35 years). All test subjects were right-handed according to a test of handedness^56^. To prevent interfering cognitions about sFST, participants were told that they would participate in an experiment concerning memory effects of haptic exploration. After participants finished the experiment, the goal of the study was unmasked and participants received 10€/h. All participants gave written informed consent. The study was approved by the Ethics Committee of University of Leipzig, Medical Faculty. The procedures used in this study adhered to the tenets of the Declaration of Helsinki.

### Experimental design

**Figure 1:**
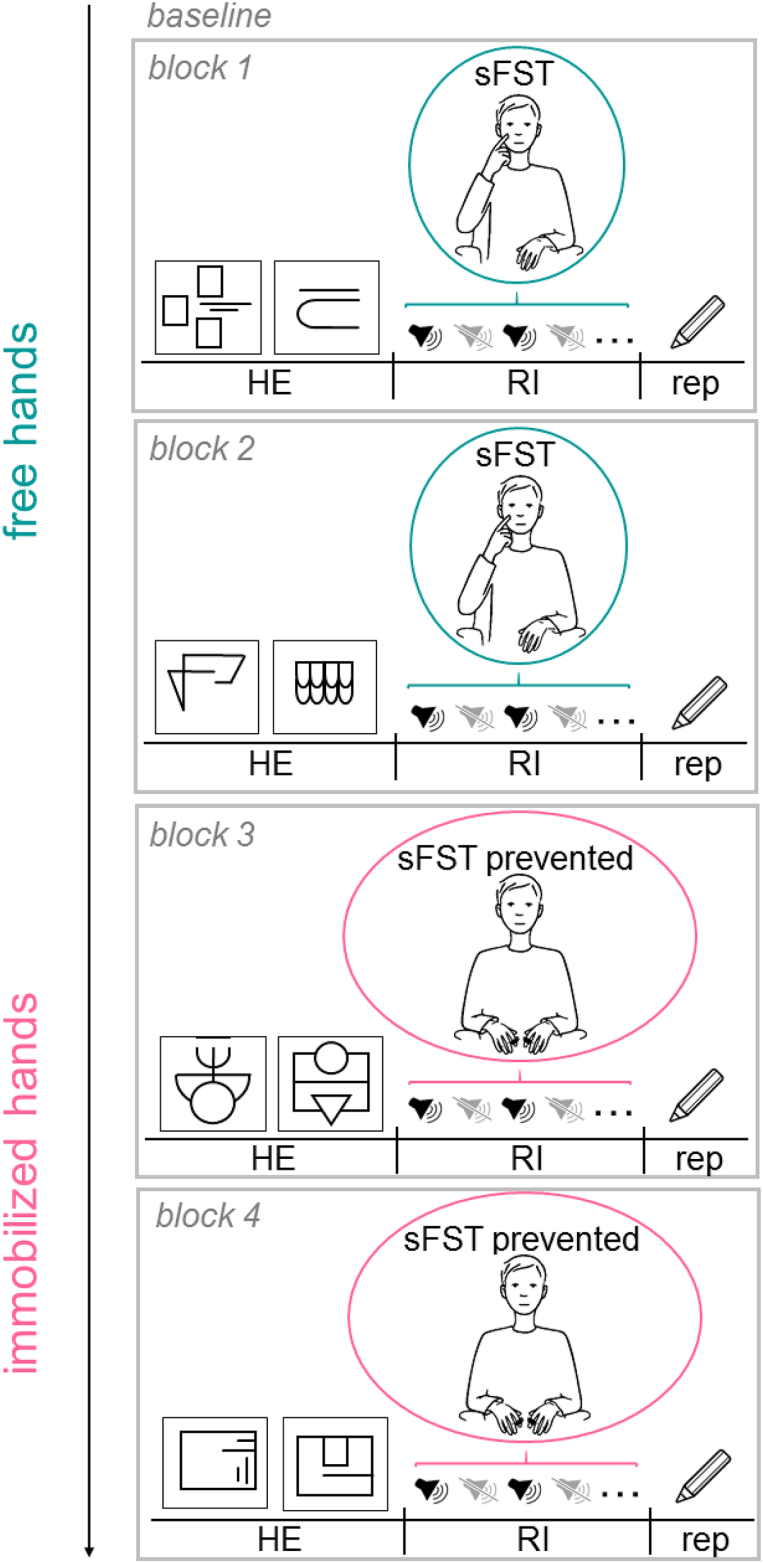
Schematic representation of the course of the experiment. After three minutes of rest (baseline, eyes open), two haptic reliefs had to be explored manually (HE) and subsequently remembered for a retention interval (RI) of 14 min. During the RI, a total of 40 distracting sounds alternated with 40 sound-free phases. After the RI, participants were asked to reproduce (rep) the remembered stimuli on a sheet of paper. After the first block (block 1) the procedure was repeated a second time (block 2). In the “free hands” condition (block 1&2), participants were able to exhibit spontaneous facial self-touches sFST during the RI. In the “immobilized hands” condition (block 3&4), the participants’ fingers were loosely fixed during the RI. Thus, the performance of sFST was suppressed. The order of conditions was randomized. Each block consisted of two different relief stimuli. Stimuli were randomized between blocks.

The experiment consisted of four experimental blocks. In each of the four experimental blocks, the participants had to explore two haptic stimuli (HE of sunken reliefs), remember them for a retention interval (RI) of 14 min and subsequently draw them on a piece of paper (rep). Distracting sounds (e.g. baby crying, explosion, siren) from a free database as well as from the database of International Affective Digitized Sounds (IADS-2)^57^ were presented during the RI. A detailed description of the sounds is given as supplementary material. Between the single sounds, there were sound-free phases. Within each RI, 40 sounds and 40 sound-free phases alternated with each other. Across participants, a total of 60 different sounds were played randomly. The durations of the sounds and sound-free phases varied between 6 and 13 s to prevent habituation and anticipation effects. In two of four experimental blocks, mechanical immobilization of the participants’ hands and fingers suppressed the execution of sFST. Half of the participants were randomly assigned to the first condition (free hands in blocks 1 and 2; immobilized hands in blocks 3 and 4), whereas the other half of the participants were assigned to the second condition (immobilized hands in blocks 1 and 2; free hands in blocks 3 and 4). A schematic representation of the experimental design and the sunken reliefs is presented in Fig. 1.

Participants were seated in a comfortable armchair with the holding equipment (for the haptic relief stimuli) in front of them. Before the experiment started, the procedure was explained to the participants and one example stimulus as well as three example sounds were presented. When the participant had no more questions, the experiment started with the haptic exploration task. An opaque screen obscured the participant’s hands and the stimulus from vision during exploration. Participants were allowed to explore the reliefs as long as they pleased; with one or both hands. Each sunken relief was milled into a plastic plate of 13 × 13 cm. The order of the sunken reliefs was randomized between participants. After haptic exploration, the opaque screen was removed and the retention interval began. During the experimental condition “free hands”, participants could move freely without any obstructions during RI. Thus, participants were able to exhibit sFST during this condition. During the experimental condition “immobilized hands”, participants’ index fingers were placed on the holding equipment where they were loosely fixed with hook and loose fastener. Thus, the participants could not move their hands freely and therefore the performance of sFST was suppressed. As a cover story, participants were informed that their finger temperature would be recorded and that they should keep their fingers on the plate steadily. During the following reproduction period, participants were to draw the structure of the sunken reliefs on a sheet of paper. After reproduction, the opaque screen was reinstalled and the next two reliefs were presented.

To explore whether individual differences in personality traits correlate with sFST behavior, we used the Freiburg Personality Inventory^39^. Through the 138 items of the questionnaire 12 personality characteristics were recorded: life satisfaction (FPI-01), social orientation (FPI-02), performance orientation (FPI-03), shyness (FPI-04), irritability (FPI-05), aggression (FPI-06), stress (FPI-07), physical complaints (FPI-08), health concerns (FPI-09), openness (FPI-10), as well as 2 secondary factors extraversion (FPI-E) and emotionality (FPI-N). Higher scores do represent higher expression of the items. The questionnaire was applied at the end of the experiment.

Facial self-touches were measured via EMG (two electrodes placed on the dorsal sides of both the left and right forearm above m. extensor carpi ulnaris) and analogous, tri-axial acceleration sensors (ADXL335; attached to the wrist of the participants). The whole experiment was videotaped through a one-way mirror. The recording system (IT-med GmbH, Germany) allowed for parallel, synchronized recording of EMG, accelerations sensors and videos of the whole experimental session with a recording rate of 256 data points per second. EEG was also recorded but the results will be presented elsewhere.

### Data analysis

The present study examined performance outcomes depending on whether participants were able to move their hands freely and perform sFST during RI or whether their hands were immobilized, thus preventing them from executing sFST. To define the type of sFST even more strictly, all self-touches of the hair, head, neck and ears were excluded as well as all sFST with obvious instrumental value (yawning, scratching, nose picking etc.). Even though sFST occurred during all experimental phases, the main analytical emphasis will be on sFST that occurred during RI as we aim to investigate the association between sFST and the maintenance of items in working memory.

The quality of the participants’ graphic reproduction of the haptic stimuli was assessed by three independent raters (S.M., M.G. and N.S). The performance outcome was evaluated using a 4-point rating scale with score 1 representing the best outcome (The drawing fully reproduces the overall structure of the target stimulus) and score 4 representing the worst outcome (The drawing does not reproduce the target stimulus). Kendall’s Concordance Coefficient (Kendall’s W) was used to assess the interrater reliability of the behavioral performance ratings of the participants. Interrater reliability for performance outcome was very good (Kendall’s *W* = 0.895, *p* < 0.001).

All statistical analyses were conducted using SPSS for Windows (version 27.0). Alpha was set at 5%. Within subject comparisons were performed via non-parametric Wilcoxon signed-rank tests. Effect sizes for the Wilcoxon signed-rank tests were calculated as r = z/√N, where z is the z-score produced by Wilcoxon signed-rank test and N is the sample size. An effect size score of 0.1 indicates a small, 0.3 a medium, and ≥ 0.5 a large effect^58^. Independent samples t-Test were used for independent group comparisons. When the assumptions of an independent samples t-Test were not met, non-parametric Mann–Whitney-U test was applied.

The data of the current study are available from the corresponding author upon request.

### Ethics approval

This study was performed in line with the principles of the Declaration of Helsinki. Approval was granted by the Ethics Committee of University of Leipzig Medical Faculty.

## Results

### Descriptive statistics

Over the entire course of the experiment, 36 of the 49 participants exhibited at least one sFST during the “free hands” condition of RI. Within these 36 participants, the number of individual sFST ranged from 1 to 14. Seven participants (19.4% of the 36 participants who showed sFST during the RI) exhibited only one sFST during RI and therefore constituted the group of low self-touching individuals (LT). Eight participants (22.4% of the 36 participants who showed sFST during the RI) exhibited an average of *Mdn* = 9.50 (range 7–14) sFST during RI and therefore constituted the group of high self-touching individuals (HT). The difference in sFST frequency during RI between the two groups was statistically significant (*U* = 0.000, *p* < 0.001). Combining all other experimental phases (baseline, haptic exploration, reproduction), HT showed considerably more sFST than LT, however, this result failed to reach significance (HT: *Mdn* = 6; range 0–9; LT: *Mdn* = 1; range 0–3; *U* = 11.000, *p* = 0.054). The order of experimental conditions (“free hands” vs. “immobilized hands”) did not affect the frequency of sFST (*U* = 235.000, *p* = 0.302). Also, performance outcomes did not differ depending on whether individuals were first assigned to the “immobilized hands” or “free hands” condition (*U* = 245.000, *p* = 0.411).

### Freiburg personality inventory

Contrary to hypothesis 1, HT and LT did not differ in any scale of the FPI-R. However, in the scales irritability (FPI-05), aggression (FPI-06), stress (FPI-07) and emotionality (FPI-N), HT tended to show higher scores than LT, but these differences failed to reach significance (Table 1).

**Table 1:** Hypothesis 1: comparisons of the FPI-R subscales between high and low self-touching individuals.

	HT		LT				95% CI	
	*M*	*SD*	*M*	*SD*	*t(13)*	*p*	Lower	Upper
FPI-01	4.13	1.246	4.86	1.676	0.969	.350	− 0.901	2.365
FPI-02	6.00	1.512	6.86	1.574	1.075	.302	− 0.865	2.580
FPI-03	4.13	1.959	4.29	1.254	0.186	.855	− 1.708	2.029
FPI-04	5.13	1.727	6.00	2.00	0.910	.379	− 1.202	2.952
FPI-05	4.75	1.282	3.86	1.464	− 1.260	.230	− 2.423	0.638
FPI-06	4.25	1.389	3.29	1.380	− 1.345	.201	− 2.513	0.584
FPI-07	4.38	0.916	3.43	1.134	− 1.789	.097	− 2.090	0.197
FPI-08	4.50	1.069	4.86	1.574	0.520	.612	− 1.125	1.840
FPI-09	4.13	1.808	4.14	2.116	0.018	.986	− 2.169	2.205
FPI-10	5.50	2.070	5.43	0.535	− 0.094	.927	− 1.820	1.678
FPI-E	4.38	1.685	4.43	1.618	0.063	.951	− 1.796	1.904
FPI-N	5.38	1.188	4.43	1.188	− 1.138	.276	− 2.744	0.851

Results of independent samples t-test.

FPI-R, Freiburg personality inventory; HT, high self-touching individuals; LT, low self-touching individuals; CI, confidence interval.

### Memory performance

**Figure 2:**
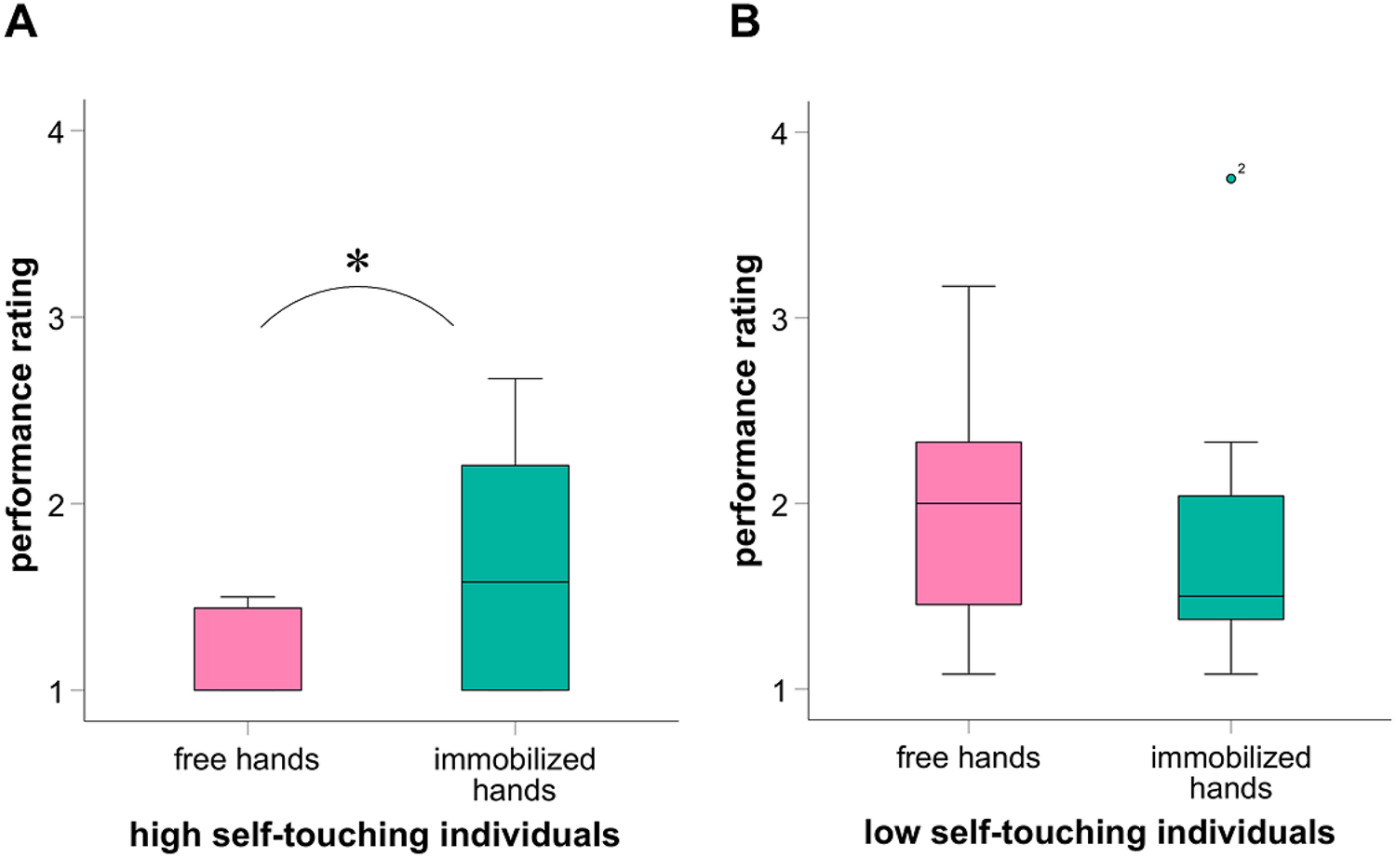
Performance ratings according to different experimental conditions (within-subjects). Score 1 represents the best outcome, score 4 represents the worst outcome. During “free hands” participants could move freely and perform spontaneous facial self-touches (sFST). During “immobilized hands” the performance of sFST was suppressed. Wilcoxon signed-ranked tests revealed that high self-touching individuals showed poorer performance outcomes when sFST were suppressed. *p < .05.

As expected in hypothesis 2, within subjects analysis revealed that HT showed significantly better performance outcomes when they were allowed to exhibit sFST during RI (*Mdn* = 1; range 1.00–1.50) compared to when the execution of sFST was suppressed during RI (*Mdn* = 1.58; range 1.00–2.67; *z* = -2.03, *p* = 0.031, *n* = 8; Fig. 2A). The effect size is *r* = 0.72 and represents a strong effect.

For LT, within-subjects analysis did not reveal any difference in the performance outcomes between the two conditions “free hands” (*Mdn* = 2; range 1.08–3.17) and “immobilized hands” (*Mdn* = 1.5; range 1.08–3.75; *z* = − 0.51, *p* = 0.344, *n* = 7; Fig. 2B), as expected in hypothesis 3.

## Discussion

The present study was the first to examine personality traits between individuals who frequently perform spontaneous facial self-touches and individuals who rarely perform sFST. Contrary to expectations, we found no significant differences in personality traits between HT and LT. We further investigated the association of mechanical suppression of sFST during the retention interval of a haptic working memory task with performance outcomes in HT and LT. Our results support previous assumptions that sFST are involved in the regulation of attentional and working memory processes and not merely represent displacement activities. A significant negative relationship was found between suppression of sFST and performance outcomes in HT, whereas suppression of sFST in LT was not significantly related to performance outcomes.

### Personality traits of high and low self-touching individuals

Contrary to hypothesis 1, HT and LT did not differ significantly in the scales of the FPI-R. However, in the scales stress and emotionality, HT tended to score higher than LT. Individuals who score high on these scales tend to be more sensitive and anxious and are likely to experience high levels of tension, which can result in nervousness and perceived stress. In addition, the analyses revealed that HT—compared to LT—tended to have higher scores in the subscales irritability and aggression. These subscales capture personality traits characterized by excitable, irritable, and unrestrained behavior. Given the small sample size, it would be too early to attribute specific personality traits to HT and LT. Nevertheless, the present findings partially reflect previous results from observational studies. Findings on sFST showed that feelings of tension and uncertainty were correlated with a higher incidence of sFST^18,59^. Moreover, recent research found a positive association between trait anxiety (as measured by the State–Trait Anxiety Inventory, STAI) and sFST frequency^30,44,60^ . Items of the scales irritability, stress and emotionality also partially mirror items of the trait anxiety of the STAI. The present findings therefore support the hypothesis that individuals who perform sFST more frequently tend to experience anxiety-related emotions. Future studies on sFST should apply the STAI to confirm the hypothesis that individuals with high trait anxiety generally exhibit higher numbers of sFST than individuals with low trait anxiety. However, as studies have discussed associations between anxiety and other psychological constructs such as stress^61^ and aggression^62^, future studies should additionally asses each of these different variables for a better understanding of the specific determinants of sFST behavior.

The absence of significant differences between HT and LT in the other scales of the FPI-R indicates that personality traits such as performance orientation, physical complaints, health concerns, openness, and extraversion are not related to the extent of sFST frequency. However, it would be too early to conclude that HT and LT do not differ in these personality traits. Rather, other testing methods that capture personality traits should be conducted on larger samples. In addition, other factors that might impact sFST behavior should be captured. Stefaniak and colleagues have suggested to examine the potential role of social factors such as education^37^. To date, it is not known whether, for example, parental sanctions about lack of table manners or the parents’ sFST behavior itself influences children's sFST behavior.

### Potential contributors to the high interindividual variability in sFST behavior

The finding that HT and LT did not differ significantly in their personality traits raises a further question: Are situational factors determining for sFST behavior or do individuals—independent of the current situation and environmental stimuli—generally show more or less pronounced sFST behavior that can be explained by factors other than trait anxiety? HT also tended to perform more sFST than LT in the other experimental phases (baseline, haptic exploration, reproduction); indicating that individual sFST behavior occurred irrespective of the experimental phase. To address the question of continuity of sFST behavior in HT and LT, temporal stability as well as cross-situational consistency of sFST behavior need to be examined. Future studies should therefore investigate whether HT execute a greater amount of sFST than LT in other situations—e.g., during non-challenging rest periods, social interaction, or manual tasks.

In addition to varying external situational factors, future research should also ascertain the state of the participants. Following the assumption that sFST are involved in the regulation of emotional and cognitive processes^5,35^, a possible explanation for the varying sFST behavior might be the individuals' current mental state. It may be that a person in a balanced state of mind has fewer regulatory needs and therefore exhibits less sFST. Previous research findings on nonverbal behavior have observed a positive relationship between self-touch and depression^63–65^. Findings from clinical patients may be an indication that a person's current mental state—irrespective of environmental circumstances or personality traits—affects sFST behavior.

### The suppression of sFST is negatively related to the performance of high self-touching individuals

In accordance with hypothesis 2, we showed that mechanical suppression of sFST in HT was related to poorer performance outcomes in a haptic memory task compared to when HT were able to exhibit sFST. Previous findings provided evidence that higher numbers of sFST are related to better performance in attention and working memory tasks^41^. The observation that sFST suppression in HT was related to poorer performance outcomes supports the hypothesis that sFST serve to regulate attentional and working memory processes^5,6,35,41^. Future studies should conduct attention tasks without additional working memory load, in order to make a clearer distinction between the regulation of attentional and working memory processes by sFST.

Moreover, it remains to be identified whether functions of emotion regulation, which have been discussed in the context of sFST^5,35^, are likewise negatively affected by the suppression of sFST. This question is particularly relevant to everyday life of anxious people who exhibit elevated sFST behaviors. Studies that have found a significant link between the frequency of sFST and trait anxiety^30,44,60^ did not address the possible behavioral as well as neurobiological functions of sFST in anxious individuals. On the one hand, Carrillo-Diaz and colleagues described sFST as "motor expression of anxiety"; on the other hand, they attributed emotion-regulating functions to sFST^44^. Observational studies that found increased sFST behavior in infants in response to emotionally stressful situations similarly discussed sFST as infancy self-comforting behavior^31,66,67^. It remains unanswered whether frequent sFST behavior actually reduces anxiety, or whether sFST behavior is no more than a physical manifestation of trait anxiety. For this purpose, studies should determine whether the ability of self-regulation in an emotionally demanding situation is impaired when the execution of sFST is suppressed.

To support the assumption of emotion-regulating functions of sFST, future investigations could—in addition to behavioral data or EEG analyses—record biological parameters such as heart rate or skin conductance. Previous literature relates increases in autonomic activity to increased emotional arousal^68–70^. A recent study examined the relationship of active self-soothing touch and cortisol responses to stress^71^. The authors found that, compared to a control group, participants providing self-soothing touch had reduced cortisol secretion responses to socio-evaluative stress. So far, it has not been investigated whether similar mechanisms are involved in spontaneous self-touch.

### Do low self-touching individuals possess alternative regulatory mechanisms?

As expected in hypothesis 3, the performance outcomes of LT, i.e., individuals who performed only one sFST during the RI, were not impaired after the mechanical suppression of sFST. This raises the question of whether LT possess alternative regulatory mechanisms that were not affected by the suppression of sFST by means of immobilized hands. So far, it is unclear whether the motor aspect of sFST (movement of the arm and hands toward the face), the sensory aspect (contact between finger and facial skin), or an interaction of motor and sensory aspects is central to the hypothesized regulatory functions of sFST^6,35^. Low self-touching individuals may perform compensatory motor actions, such as straightening the upper body, moving the feet, or changing the sitting position, which were not recorded in the present study. Future studies should therefore capture body movements that do not result in a touch event to determine whether they occur more frequently during sFST suppression.

### Spontaneous self-touches of other body parts

In the present study, we focused on spontaneous touches of the own face, since they occur more often than spontaneous touches of other body parts^29,59^. So far, no studies have addressed why the face is such a predominant goal. The proximity of the cortical representation of the hand and face might be related to the overall high frequency of sFST compared to self-touches of other body parts^13^. Furthermore, the potential role of the facial nerve as well as the trigeminal nerve, which is part of the facial feedback of emotional expression^72^, is yet unclear^6^. Nevertheless, it would be insightful to ascertain whether HT and LT also differ in terms of spontaneous self-touches to other body parts. For example, fidgeting (hand-to-hand behavior) has been theoretically related to self-regulation^73,74^. The same holds true for other self-directed behaviors that have also been observed in primates^75–77^. In this context, another unresolved question is whether continuous touches such as stroking, which can last up to 100 seconds^74^, have a different function than short touches such as sFST, which have been reported to last shorter than 3 seconds^13^. Future studies should therefore further investigate the relationship between sFST and other self-touch behaviors, quantifying their influence on performance outcomes and emotion regulation.

### Limitations and future directions

Our study was the first to address differences between individuals exhibiting frequent (HT) or low (LT) sFST behavior. Because no study has made this distinction so far, we have not been able to refer to an existing cutoff value that defines individuals as HT or LT. Studying spontaneous behaviors within controlled experimental trials is a difficult endeavor since spontaneous behaviors are not strictly predictable and may only be provoked to a limited extent in the context of an experiment. It is therefore difficult to predict the number of sFST per participant. The present study results may provide a cutoff value for following studies to classify individuals as either HT or LT.

The present study investigated behavioral consequences of sFST suppression in HT and LT. For this purpose, sFST were suppressed during retention intervals by loosely fixating the participants' index fingers with hook and loose fastener. This experimental condition represents a persistent sensory stimulation of the index fingers. We cannot exclude the possibility that the persistent sensory stimulation of the fingers may have an impact on the participants' attention focus or regulatory mechanisms. Therefore, to prevent the potential impact of sensory stimulation, future studies should use other methods to suppress sFST. For example, a shield could be installed above the participants’ shoulders, preventing the face from being touched by the own hands.

The finding that suppression of sFST has differential behavioral consequences on HT and LT should provide an incentive to distinguish between these two groups in future studies on sFST. Regarding respiratory infection transmission through sFST, the appropriateness of suppressing sFST using methods such as smart wearable devices should be critically examined. Moreover, researchers discussed the volitional suppression of sFST using behavioral strategies such as awareness training^78^. However, the active and intentional suppression of sFST might require additional cognitive resources^13^. Thus, it should be addressed whether HT experience even greater cognitive performance impairments due to additional mental effort during the volitional suppression of sFST. Alternatives that do not entirely suppress sFST behavior, such as better hand hygiene, should be focused to ensure that individuals who exhibit frequent sFST behavior do not experience negative consequences associated with the suppression of sFST.

## Conclusion

Consistent with our expectations, we found that suppression of sFST has different behavioral consequences depending on whether individuals exhibit frequent or infrequent sFST behavior. Individuals who frequently perform sFST show poorer performance outcomes when sFST were suppressed during the retention interval of a working memory task. High and low self-touching individuals do not seem to differ in personality variables measured by the FPI-R, which raises several questions: Why do certain individuals exhibit frequent sFST behavior, while others rarely perform sFST? Is sFST behavior a stable behavioral trait or are situational factors determining the frequency of sFST? Future studies should consider possible causes, personality variables, contextual factors as well as functions of sFST in a more differentiated way.

## Supplementary Information

Supplementary Information.

## Data Availability

All data are available upon request to the corresponding author. The experiment was not preregistered.
