# Supplementary material for: The suppression of spontaneous face touch and resulting consequences on memory performance of high and low self-touching individuals
Source: Sci Rep. 2022 May 23;12:8637. doi: 10.1038/s41598-022-12044-4 (PMC9125538; doi:10.1038/s41598-022-12044-4)
Supplement: Supplementary file 1 — Supplementary Information. [file 41598_2022_12044_MOESM1_ESM.docx]

**Supplementary Material**

Description of the sound material:

The stimulus material consisted of a total of 60 sounds. 46 sounds were taken from the International Affective Digitized Sounds Database (2nd Edition; IADS-2). The stimuli are standardized, emotionally evocative sounds that cover a wide range of semantic categories. In a normative study by Bradley and colleagues, the affective valence of the stimuli was rated by a total of 100 participants on a 9-point rating scale (Bradley et al., 2007). Ratings were scored such that 9 represents a high rating (high pleasure) and 1 represents a low rating (low pleasure). We chose stimuli whose valence was rated as low pleasure (M = 3.21, SD = 1.07), e.g. baby crying, explosion or siren. Additionally, we chose another 14 sounds from a free audio database (https://freesound.org) that we judged as unpleasant, e.g. dentist drill or jackhammer. All sounds are available on the website of the Haptic Research Laboratory (https://haptiklabor.medizin.uni-leipzig.de) under “Research” > “Aversive Sounds for sFTG”.

Sound numbers IADS-2:

100, 105, 106, 115, 116, 130, 133, 251, 252, 261, 276, 277, 278, 279, 280, 285, 286, 287, 290, 291, 292, 310, 319, 322, 380, 403, 420, 422, 423, 424, 500, 501, 502, 600, 625, 626, 698, 699, 702, 706, 708, 709, 711, 712, 723, 730
